# Effects of increasing doses of trenbolone acetate and estradiol on finishing phase growth performance, carcass trait responses, and serum metabolites in beef steers following implantation

**DOI:** 10.1093/tas/txaa158

**Published:** 2020-08-26

**Authors:** Dathan T Smerchek, Zachary K Smith

**Affiliations:** Department of Animal Science, South Dakota State University, Brookings, SD

**Keywords:** estradiol, growth performance, implant, trenbolone acetate

## Abstract

Yearling Simmental × Angus crossbred beef steers (*n* = 240; allotment BW = 365 ± 22.5 kg) from a South Dakota auction facility were transported 117 km to Brookings, SD and used in a randomized complete block design feedlot study to evaluate the effects of implants (both from Zoetis, Parsippany, NJ) containing increasing doses of trenbolone acetate (TBA) and estradiol benzoate (EB) administered 124 d prior to harvest have on finishing phase growth performance, carcass characteristics, and serum concentrations of urea-N (SUN) and insulin-like growth factor I (IGF-I). Thirty pens (10 pens/treatment) were assigned to 1 of 3 treatments: 1) negative control given no implant (NI); 2) a steroidal implant containing 100 mg TBA and 14 mg EB administered subcutaneously in the center one-third of the ear on day 1 (Synovex Choice, Zoetis, Parsippany, NJ; CH); 3) a steroidal implant containing 200 mg TBA and 28 mg EB administered subcutaneously in the center one-third of the ear on day 1 (Synovex Plus, Zoetis; PL). Cattle were fed for 124 d post-implantation. Steers were fed a common diet throughout the study. Treatment effects were evaluated by the use of orthogonal polynomials. Pen was the experimental unit for all analyses; an α of 0.05 determined significance. There was a quadratic effect (*P* = 0.01) on carcass-adjusted final BW. Increasing doses of TBA and EB resulted in a linear increase for both average daily gain (*P* = 0.01) and dry matter intake (*P =* 0.02). A quadratic effect on gain-to-feed ratio was observed (*P* = 0.01). No quadratic (*P* ≥ 0.40) or linear (*P* ≥ 0.14) effects were observed for dressing percentage, rib fat (RF), calculated yield grade, or marbling scores. A quadratic increase (*P* = 0.01) in hot carcass weight (HCW) and a linear increase (*P* = 0.01) in ribeye area (REA) were detected. No significant implant × day interaction (*P* ≥ 0.09) was noted for serum concentrations of urea-N or IGF-I. Implants decreased (*P* = 0.01) SUN compared with NI. Serum concentration of IGF-I was increased (*P* = 0.04) in implanted steers compared with NI steers. In yearling crossbred beef steers, the use of steroidal implants containing a combination of 100 mg TBA + 14 mg EB or 200 mg TBA + 28 mg EB increases growth performance, HCW, and REA at equal RF accumulation without detriment to marbling score compared with nonimplanted steers.

## INTRODUCTION

Steroidal implants have been used in U.S. commercial beef production for over 63 yr and can be expected to improve growth rate 10% to 30%, feed efﬁciency 5% to 15%, and carcass leanness 5% to 8% (Preston, 1999). A meta-analysis investigating feedlot steer implant programs found in a comparison that across all single-implant treatments, implants increase live weight gain, dry matter intake (DMI), dressing percentage (DP), hot carcass weight (HCW), ribeye area (REA), and gain-to-feed ratio (G:F) and decrease the percentage of carcasses grading USDA Choice or greater, and USDA marbling score compared with nonimplanted steers (Reinhardt and Wagner, 2014). Effect of steroidal implant on marbling score is often shown to be negative; however, it has been reported (Johnson et al., 1996a) that administration of a combination trenbolone acetate (TBA) and estradiol-17β (E_2_) implant did not have deleterious effects on marbling score.

The androgenic constituent of steroidal implants, TBA, has a direct effect on skeletal muscle that increases muscle tissue anabolism while decreasing muscle tissue catabolism, thus increasing net protein synthesis (Smith and Johnson, 2020). Previous research has shown that the anabolic effect of steroidal implants results in decreased serum concentration of urea-N (SUN) concentrations after implantation with a combination TBA + E_2_ implant (Smith et al., 2018). The estrogenic constituent of steroidal implants, E_2_, functions by increasing production and release of hepatic somatotropin and IGF-I (Reinhardt, 2007) and has been reported to increase local IGF-I production in steers through measurement of concentration of IGF-I mRNA in the longissimus muscle of steers implanted with a combination TBA + E_2_ implant (Johnson et al., 1998). It has been previously reported that combination TBA + E_2_ implants increase circulating serum concentration of IGF-I (Johnson et al., 1996a; Smith et al., 2018). It has been demonstrated that increasing the initial dosage of hormonal constituents does not increase cumulative live growth performance (Hilscher et al., 2016) when steers and heifers were administered the same terminal implant. Others have indicated in heifers that a greater total dose of steroidal hormones does not increase live-basis growth performance and only moderately increases HCW as well as indicators of carcass muscularity and carcass leanness (Smith et al., 2020).

The objective of this study was to evaluate the effects of increasing doses of TBA and estradiol benzoate (EB) on finishing phase growth performance, carcass characteristics, and serum concentration of urea-N and IGF-I. The hypothesis was that increasing terminal implant dosage in steers would increase carcass-adjusted growth performance, HCW, and muscularity.

## MATERIALS ANDS METHODS

### Use of Animal Subjects

Animal care and handling procedures used in this study were approved by the South Dakota State University Animal Care and Use Committee (Approval number: 18-096A).

### Animal Description and Initial Processing

Yearling Simmental × Angus crossbred beef steers (*n* = 240; allotment BW = 365 ± 22.5 kg) were transported 117 km from a South Dakota auction facility to the Ruminant Nutrition Center (RNC) in Brookings, SD for use in this experiment. Steers were allotted to 30 concrete surface pens (7.25 × 7.25 m; 6.57 m^2^/steer; 90.6 cm of bunk space/steer; *n* = 8 steers/pen) 36 d prior to being implanted. The first 6 pen replicates began on test 14 d prior to the last 4 pen replicates due to timing of acquisition of sufficient cattle needed in order to conduct the experiment.

Initial processing included an individual body weight measurement, application of a unique identification ear tag, and a rectal temperature measurement along with vaccination for respiratory syncytial virus (BRSV), bovine rhinotracheitis (IBR), bovine viral diarrhea (BVD) Types 1 and 2, parainfluenza-3 (PI3), Mannheimia haemolytica, and clostridium perfringens type A and administered pour-on moxidectin. Cattle were re-vaccinated 36 d after initial processing for clostridium perfringens type A. Any steer with a rectal temperature of greater than 39.4 °C was administered tulathromycin according to label instructions.

### Experimental Design and Treatments

Pens were assigned to 1 of 3 implant treatments with 10 replicate pens assigned to each treatment: 1) negative control given no implant (NI); 2) a steroidal implant containing 100 mg TBA and 14 mg EB administered subcutaneously in the center one-third of the ear on day 1 (Synovex Choice, Zoetis, Parsippany, NJ; CH); 3) a steroidal implant containing 200 mg TBA and 28 mg EB administered subcutaneously in the center one-third of the ear on day 1 (Synovex Plus, Zoetis; PL).

### Dietary Management

Composition of the finishing diet fed from day 18 to harvest is presented in Table 1. Due to an evolving roughage inventory, a switch to grass hay from oatlage occurred with 12 d remaining in the experiment. The finishing diet consisted of dry-rolled corn, dried distillers grains plus solubles, and oatlage or grass hay was fed and contained 2.10 Mcal/kg of NEm, and 1.40 Mcal/kg of NEg. A liquid supplement was provided to add 30 g/907 kg of monensin sodium to diet DM along with supplemental vitamins and minerals to meet NASEM (2016) requirements.

**Table 1.** Composition of finishing diets (DM basis)

Item	Finishing diet
Dry-rolled corn, %	69.70
Dried distillers grains plus solubles, %	17.00
Oatlage, %	8.37
Liquid supplement^*a*^, %	4.93
Dry matter, %	77.50
Crude protein, %	14.20
Neutral detergent fiber, %	16.60
Acid detergent fiber, %	6.84
Ash, %	5.25
Ether extract, %	5.13
NEm^*b*^, Mcal/kg	2.10
NEg^*c*^, Mcal/kg	1.40

All values except dry matter or a DM basis.

^*a*^Liquid supplement: formulated to add 30 g/907 kg of monensin sodium to diet DM and vitamins and minerals to meet NASEM (2016) requirements.

^*b*^Net energy for maintenance.

^*c*^Net energy for gain.

All steers were fed twice daily at 0800 and 1400 h; bunks were managed according to a slick bunk management approach. When necessary, orts were collected, weighed, and dried in a forced air oven at 100 °C for 24 h to determine DM content if carryover feed went out of condition or was present on weigh days. If carryover feed was present on weigh days, the residual feed was removed prior to the collection of BW measurements. The DMI of each pen was adjusted to reflect the total DM delivered to each pen after subtracting the quantity of dry orts for each interim period.

Diets presented in Table 1 are actual DM diet composition from weekly ingredient DM analysis, actual assayed nutrient concentrations from weekly commodity ingredient sampling of the dry-rolled corn, dried distillers grains plus solubles and forage source for crude protein (CP), neutral detergent fiber (NDF; except for corn where the NDF was estimated to be 9%), acid detergent fiber (ADF; except for corn where the ADF was estimated to be 3%), ash, and ether extract (EE): method no. 968.06 (AOAC, 2016) for CP, using the Rapid Max N Exceed, Elementar, Mt. Laurel, NJ; NDF and ADF (Goering and VanSoest, 1970); method no. 942.05; (AOAC, 2012) for ash; and EE using petroleum ether, method no. 2003.06; (AOAC, 2007), and tabular energy values according to Preston (2016) were used.

### Blood Sample Collection

Whole blood samples were collected into 10-mL nonadditive tubes during the weighing process prior to feeding on days 1, 28, 56, and 84 (relative to implantation) from sentinel steers (*n* = 2 steers/pen). Whole blood was allowed to clot for 24 h at 4 °C and was subsequently centrifuged at 1250 × *g* at 4 °C for 20 min. A total of three aliquots were collected and stored at −20° C until subsequent analyses to quantify serum concentrations of urea-N and IGF-I.

### Serum Concentrations of Urea-N and Insulin-Like Growth Factor I

Serum concentrations of urea-N were determined by a method described by Fawcett and Scott (1960) using sodium phenate and sodium hypochlorite. The determination of SUN is measured based on the reaction of ammonia with sodium phenate and hypochlorite to yield a blue color to be measured in a spectrophotometer. Absorbance for reactions of standards and samples were read at 625 nm. Samples were considered for re-runs if the coefficient of variation (CV) was greater than 10% among triplicate determinations. Intra- and inter-assay CV were 6.3% and 10.9%, respectively.

Serum concentrations of insulin-like growth factor I (IGF-I) were determined in duplicate via radioimmunoassay (RIA) procedure (Echternkamp et al., 1990; Funston et al., 1995). Insulin-like growth factor binding proteins (IGFBP) in serum were extracted using a 1:17 ratio of sample to acidified ethanol (12.5% 2 N HCl: 87.5% absolute ethanol; Daughaday et al., 1980). Extracted samples were centrifuged (12,000 × *g* at 4 °C) to separate IGFBP. A portion of the resulting supernatant was removed and neutralized with 0.855 M Tris base, incubated for an additional 4 h at 4 °C, and then centrifuged at 12,000 × *g* at 4 °C to remove any additional IGFBP. When samples of this extract, equivalent to the original serum sample, were subjected to Western ligand blot analysis and subsequent phosphorimagery, no detected binding of I-IGF-I to IGFBP was observed. Inhibition curves of the neutralized extracted serum ranging from 12.5 to 50 µL were parallel to the standard curve. Recombinant human IGF-I (GF-050; Austral Biological, San Ramon, CA) was used as the standard and radioiodinated antigen. Antiserum AFP 4892898 (National Hormone and Peptide Program, National Institutes of Diabetes, Digestive and Kidney Diseases, Bethesda, MD) was used at a dilution of 1:62,500. Sensitivity of the assay was 14.7 pg/tube. Samples were considered for re-runs if the CV was greater than 10% among duplicate determinations. No samples were considered for re-runs; the RIA was completed in a single assay and the intraassay CV was 7.7%.

### Growth Performance Calculations and Carcass Data Collection

Steers were individually weighed and harvested after an average of 124 d on feed. Weight gain was based upon initial un-shrunk BW (average of day −1 and 1 BW) and final BW was calculated from HCW/0.625. All steers that were pulled from their home pen for health evaluation were then monitored in individual hospital pens prior to being returned to their home pens. When a steer was moved to a hospital pen the appropriate amount of feed from the home pen was removed and transferred to the hospital pen. If the steer in the hospital returned to their home pen, this feed remained credited to the home pen. If the steer did not return to their home pen, all feed that was delivered to the hospital pen was deducted from the feed intake record for that particular pen back to the date the steer was hospitalized.

Cattle were on feed for an average of 124 d post-implantation before being marketed and harvested at a commercial abattoir (Tyson Fresh Meats, Dakota City, NE) when the population reached sufficient fat cover to grade USDA Choice. Carcass data including HCW, REA, 12th rib fat (RF), kidney, pelvic, and heart fat percent, and USDA marbling score were collected by the camera grading system at the abattoir. Yield grade (YG) was calculated by using the USDA regression equation (USDA, 1997). Estimated empty body fat (EBF) from carcass traits was calculated according to Guiroy et al. (2002). Retail yield (RY) as percentage of HCW was calculated according to Murphey et al. (1960).

### Statistical Analysis

Growth performance and carcass data were analyzed as a randomized complete block design experiment using the GLIMMIX procedure of SAS 9.4 (SAS Inst. Inc., Cary, NC), considering implant treatment and block (pen location) as fixed effects. Pen served as the experimental unit for growth performance and carcass traits. Treatment effects were evaluated by the use of orthogonal polynomials (Steel and Torrie, 1960). All results are reported as least squares means.

Serum concentrations of urea-N and IGF-I data were analyzed according to randomized complete block design appropriate for repeated measures using the MIXED procedure of SAS 9.4 (SAS Inst. Inc.). The model included the fixed effects of implant, day, and their interaction. Day was included as the repeated variable and pen served as the experimental unit. Day 0 values for serum concentrations of urea-N and IGF-I were used as covariate adjustments (*P* ≤ 0.06) in the repeated measures model. The covariance structure with the lowest Akaike information criterion was used (Littell et al., 1998). Compound symmetry was the covariance structure used for serum concentration of urea-N and Huynh-Feldt was the covariance structure used for serum concentration of IGF-I. All results are reported as least squares means. An α of 0.05 determined significance and an α of 0.06 to 0.10 was considered a tendency.

## RESULTS

### Animal Growth Performance

Growth performance and carcass trait responses are located in Table 2. Initial body weight at the time of implant did not differ (*P* = 0.51) between treatments. A quadratic effect (*P* = 0.01) on carcass-adjusted final BW was noted; CH was increased 4.5% and PL was increased 5.6% relative to the NI control. Increasing doses of TBA and EB resulted in a linear increase (*P* = 0.01) in cumulative ADG, the increases compared with the NI control group were 18.4% and 21.6%, respectively, for CH and PL treatments. Increasing doses of TBA and EB also resulted in a linear increase in DMI (*P =* 0.02). DMI was increased by 2.3% and 7.0% for CH and PL treatments, respectively, relative to NI. A quadratic effect on G:F was observed for implanted treatments, increasing by 21.1% and 19.5% for CH and PL, respectively, compared to NI.

**Table 2.** Effect of implant on cattle performance and carcass characteristics

	Implant				Contrast *P*-value	
Item	NI	CH	PL	SEM	L	Q
Pens	10	10	10	–	–	–
DOF	124	124	124	–	–	–
Initial body weight (BW), kg	400	397	397	3.4	0.51	0.79
Final BW, kg^*a*^	553	578	584	2.5	0.01	0.01
Average daily gain (ADG), kg/d	1.25	1.48	1.52	0.022	0.01	0.10
Dry matter intake (DMI), kg/d	9.66	9.93	10.34	0.196	0.02	0.77
ADG/DMI, kg/kg	0.123	0.149	0.147	0.0030	0.01	0.01
Dressing percentage, %	62.64	62.82	62.92	0.246	0.44	0.89
Hot carcass weight (HCW), kg	346	362	365	1.67	0.01	0.01
Ribeye area, cm^2^	79.81	83.10	85.94	0.924	0.01	0.86
Rib fat, cm	1.12	1.17	1.14	0.033	0.66	0.56
Marbling^*b*^	463	458	447	10.4	0.28	0.83
Estimated empty body fat, %^*c*^	28.64	28.71	28.52	0.205	0.70	0.61
Calculated yield grade	2.92	2.92	2.79	0.062	0.14	0.40
Retail yield, %^*d*^	50.62	50.64	50.92	0.142	0.15	0.45

Treatments: 1) negative control given no implant (NI); a steroidal implant containing 100 mg TBA and 14 mg EB administered subcutaneously in the center one-third of the ear on day 1 (Synovex Choice, Zoetis, Parsippany, NJ; CH); a steroidal implant containing 200 mg TBA and 28 mg estradiol benzoate administered subcutaneously in the center one-third of the ear on day 1 (Synovex Plus, Zoetis; PL).

^*a*^Calculated from HCW/0.625.

^*b*^400 = Small^00^ (USDA Low Choice).

^*c*^According to Guiroy et al. (2002).

^*d*^As a percentage of HCW according to Murphey et al. (1960).

### Carcass Characteristics

No linear (*P* ≥ 0.14) or quadratic (*P* ≥ 0.40) effects were observed for DP, RF, YG, or USDA marbling scores. However, a quadratic increase (*P* = 0.01) in HCW was noted. HCW was increased by 4.6% and 5.5% for CH and PL, respectively, compared with NI. A linear increase (*P* = 0.01) in REA was observed. Ribeye area was increased by 4.1% and 7.7% for CH and PL treatments, respectively, compared with NI steers.

### Serum Concentrations of Urea-N and Insulin-Like Growth Factor I

**Figure 1.**
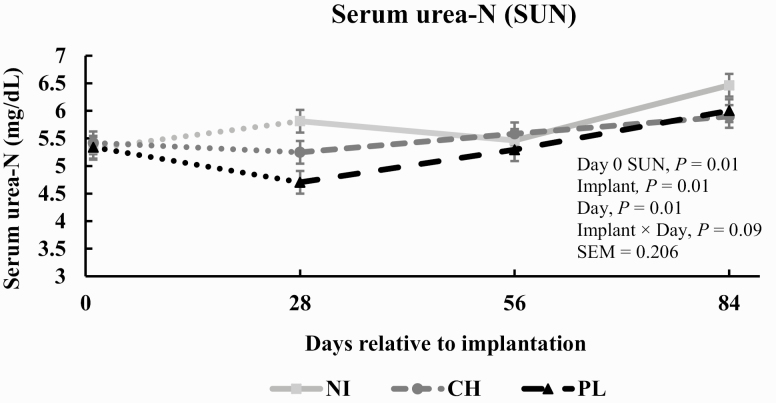
Effect of implant treatment on serum concentration of urea-N (SUN) in finishing steers (*n* = 10 pens/treatment; pooled implant × day SEM = 0.206). Day 0 SUN values were included as a covariate (*P* = 0.01) in the model. Treatments were: 1) negative control given NI; 2) a steroidal implant containing 100 mg TBA and 14 mg EB administered subcutaneously in the center one-third of the ear on day 1 (Synovex Choice, Zoetis, Parsippany, NJ; CH); 3) a steroidal implant containing 200 mg TBA and 28 mg estradiol benzoate administered subcutaneously in the center one-third of the ear on day 1 (Synovex Plus, Zoetis; PL).

**Figure 2.**
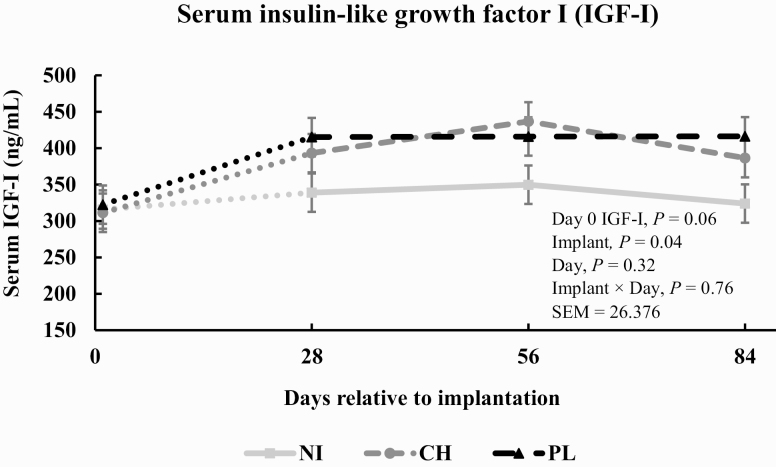
Effect of implant treatment on serum concentration of insulin-like growth factor I (IGF-I) in finishing steers (*n* = 10 pens/treatment; pooled implant × day SEM = 26.376). Day 0 IGF-I values were included as a covariate (*P* = 0.06) in the model. Treatments were: 1) negative control given NI; 2) a steroidal implant containing 100 mg TBA and 14 mg EB administered subcutaneously in the center one-third of the ear on day 1 (Synovex Choice, Zoetis, Parsippany, NJ; CH); 3) a steroidal implant containing 200 mg TBA and 28 mg EB administered subcutaneously in the center one-third of the ear on day 1 (Synovex Plus, Zoetis; PL).

A significant implant × day interaction (*P* = 0.09) was not noted for serum concentrations of urea-N (Figure 1). The main effect of implant decreased (*P* = 0.01) serum concentrations of urea-N. Steers from CH tended (*P* = 0.07) to have decreased serum concentrations of urea-N compared with NI by 5.8% and steers from PL had decreased (*P* = 0.01) serum concentration of urea-N compared with NI by 9.8%. Serum concentration of urea-N increased (*P* = 0.01) as days post-implantation increased. NI × day interaction (*P* = 0.76) was detected for concentrations of serum IGF-I (Figure 2). However, the main effect of implant increased (*P* = 0.04) serum concentrations of IGF-I. Steers from CH had increased (*P* = 0.04) serum concentration of IGF-I by 20.1% compared with NI steers; steers from PL had increased (*P* = 0.02) serum concentration of IGF-I by 23.2% compared with NI steers. Serum concentration of IGF-I was not influenced by days post-implantation (*P* = 0.01).

## DISCUSSION

### Animal Growth Performance

Increasing doses of TBA and EB from 100 mg TBA + 14 mg EB (CH) to 200 mg TBA + 28 mg EB (PL) resulted in a linear increase in cumulative ADG. These results agree well with previously reported findings regarding gain responses for cattle following implantation with a single androgenic + estrogenic combination implant (Duckett et al., 1997; Johnson and Beckett, 2014). In the present study, DMI increased linearly with increasing doses of TBA and EB. Increased DMI due to exposure to a combination androgenic + estrogenic implant also concurred with previous research findings (Duckett et al., 1997; Reinhardt and Wagner, 2014; Smith et al., 2018). Increases in DMI as a result of anabolic implant exposure are likely linked to concurrent increases in final BW (Guiroy et al., 2002). However, in the present study, there was a quadratic effect on carcass-adjusted final BW; CH was increased 4.5% and PL increased 5.6% relative to the NI control group. In the present study, the highest dose of TBA and EB (PL) did not result in increased performance relative to the CH treatment. Therefore, the linear increase of DMI as a response to increasing levels of TBA and EB may not be so simply explained as a result of increasing final BW due to exposure to a more potent terminal implant. Use of a terminal implant, in the present study, caused a quadratic effect on G:F, increasing by 21.1% and 19.5% for CH and PL treatments, respectively, compared with NI steers. This positive response in gain efficiency following administration of a terminal implant is in agreement with reported information from a meta-analysis by Wileman et al. (2009) as well as a number of other analyses (Duckett et al., 1997; Reinhardt, 2007; Johnson and Beckett, 2014) in which single-implant protocols were compared against a nonimplanted control treatment.

### Carcass Characteristics

In the present study, use of a combination TBA + EB implant did not influence DP which is similar with previously reported information using TBA + E_2_ (Duckett et al., 1997). It has been well documented that the use of combination TBA + E_2_ implants in steers results in a significant increase in HCW relative to nonimplanted steers (Bartle et al., 1992; Duckett et al., 1997; Pritchard, 2000; Smith et al., 2018). Implants increase the amount of protein deposition and decrease the amount of fat deposition at a given weight, thus causing implanted animals to reach similar body composition to that of a nonimplanted animal at a heavier weight; thus, the increase in HCW occurs concurrently with increases in live BW. In the present study, increasing doses of TBA + EB from 100 mg TBA + 14 mg EB (CH) to 200 mg TBA + 28 mg EB (PL) did not result in additional HCW between the two implants.

Reduced marbling score and corresponding lowered quality grades have long been a concern when using combination TBA + E_2_ terminal implants. Reduced or delayed subcutaneous and intramuscular fat deposition often occurs in implanted steers fed for equal days due to a shift in composition of gain (Smith et al., 2018), and also, as reported by Smith et al. (2017), a decrease in expression of important adipogenic genes in the skeletal muscle of steers due to exposure to combination TBA + E_2_ implant. It is then of interest, in the present study, that use of combination TBA + EB terminal implant of differing doses did not result in a significant decrease in marbling score compared with NI controls. This agrees with findings from Johnson et al. (1996a), but runs counter to a considerable volume of previous work which has indicated that use of a combination TBA + E_2_ implant results in decreased marbling score (Duckett et al., 1997; Pritchard, 2000; Smith et al., 2018). Bruns et al. (2005) reported that excessive anabolic exposure at key growth stages can have a detrimental impact marbling deposition in beef steers. The level of anabolic exposure experienced by steers from both CH and PL treatments was likely not excessive as evidenced by the lack of an impact on USDA marbling score following implantation with TBA + EB implant. Use of steroidal implants containing a combination of TBA and EB increased HCW, and REA at equal RF accumulation without detriment to USDA marbling score.

### Serum Concentrations of Urea-N and Insulin-Like Growth Factor I

Serum concentration of urea-N did not differ at the time of implantation. Serum concentration of urea-N decreased following implantation and this is consistent with work from (Parr et al., 2014; Smith et al., 2018). In the present study, implantation with 100 mg or 200 mg of TBA and 14 mg or 28 mg of EB resulted in an increase in serum concentration of IGF-I which is consistent with other findings (Johnson et al., 1996b; Bryant et al., 2010; Parr et al., 2014; Smith et al., 2018; Smith et al., 2019). Serum concentration of IGF-I did not increase as days on feed increased which is inconsistent with what others have demonstrated (Johnson et al., 1996a; Bryant et al., 2010; Parr et al., 2014; Smith et al., 2019). An anticipated increase in anabolism occurred following administration of a TBA + EB implant and can be identified by a reduction in serum concentration of urea-N following implantation; this coupled with a simultaneous increase in serum concentration of IGF-I aligns well with what has been demonstrated previously in beef steers (Johnson et al., 1996a; Smith et al., 2018).

## CONCLUSIONS

In yearling crossbred beef steers harvested 124 d post-implantation, the use of steroidal implants containing a combination of 100 mg TBA + 14 EB or 200 mg TBA + 28 EB increases final BW, ADG, DMI, gain efficiency, HCW, and REA at equal RF accumulation without detriment to marbling score compared with nonimplanted steers. Use of TBA and EB combination implants, in this study, resulted in increased anabolism as suggested by the observed reduction in serum concentration of urea-N and increased serum concentration of IGF-I compared with NI steers. These results indicate that the use of a lower dose implant containing 100 mg TBA + 14 mg EB can result in comparable growth performance to an implant containing 200 mg TBA + 28 mg EB. Additionally, these results provide further evidence that one can capture carcass trait related benefits that TBA + EB implants offer without detriment to marbling score.
